# The factors associated with transient hypothyroxinemia of prematurity

**DOI:** 10.1186/s12887-021-02826-6

**Published:** 2021-08-14

**Authors:** Aslan Yilmaz, Yavuz Ozer, Nesrin Kaya, Hande Turan, Hazal Cansu Acar, Oya Ercan, Yildiz Perk, Olcay Evliyaoglu, Mehmet Vural

**Affiliations:** 1 Department of Neonatology, Cerrahpasa Faculty of Medicine, Istanbul University-Cerrahpasa, Kocamustafapasa, Fatih, 34098 Istanbul, Turkey; 2 Department of Pediatric Endocrinology, Cerrahpasa Faculty of Medicine, Istanbul University-Cerrahpasa, Kocamustafapasa, Fatih, 34098 Istanbul, Turkey; 3 Department of Public Health, Cerrahpasa Faculty of Medicine, Istanbul University-Cerrahpasa, Kocamustafapasa, Fatih, 34098 Istanbul, Turkey

**Keywords:** Newborn, Preterm, Hypothyroxinemia, Thyroid, Small for gestational age, Congenital heart disease

## Abstract

**Background:**

Hypothyroxinemia is defined by low levels of thyroxine (T4) despite low or normal levels of thyroid-stimulating hormone (TSH). This study aimed to evaluate the factors associated with transient hypothyroxinemia of prematurity (THOP) in newborns admitted to the neonatal intensive care unit (NICU).

**Method:**

This is a single center, retrospective, case-control study. Premature newborns, between 24 and 34 weeks of gestation, hospitalised between January 2014–December 2019 in Istanbul University-Cerrahpasa Faculty of Medicine NICU were analyzed through their medical records. Thyroid function tests were routinely performed between the 10th and 20th days of postnatal life and were evaluated according to the gestational age references. Thirty six possible associated factors (prenatal and postnatal parameters, medical treatments, clinical diagnoses and applications in NICU) were searched in the patient group with THOP (*n* = 71) and the control group with euthyroid prematures (*n* = 73). The factors for THOP were identified by univariate analysis, followed by multivariate analysis.

**Results:**

Mean gestational ages of the study and the control groups were 29.7 ± 2.48 and 30.5 ± 2.30 weeks, respectively (*p* = 0.606). The birth weight, small for gestational age (SGA), intraventricular hemorrhage (IVH), congenital heart disease (CHD) were found to be the possible associated factors for THOP in the univariate analysis and CHD (*p* = 0.007, odds ratio [OR]:4.9, 95% confidence interval [CI]: 1.5–15.8), BW (*p* = 0.004, OR:0.999, 95% CI: 0.9–1.0) and SGA (*p* = 0.010, OR:4.6, 95% CI: 1.4–14.7) were found to be factors associated with THOP determined by univariate logistic regression analysis.

**Conclusıons:**

Although some treatment practices might have had direct effects on pituitary–thyroid axis, related with the severity of the newborn clinical conditions, non of them was found to be a associated factor for THOP. However, CHD and SGA may be considered as associated factors with THOP detected in preterm infants.

## Background

Transient hypothyroxinemia of prematurity (THOP) is defined by low levels of thyroxine (T4) despite low or normal levels of thyroid-stimulating hormone (TSH) [1]. Hypothyroxinemia is observed in around 50% of premature newborns and its risk increases as the gestational week decreases [2, 3]. Serum T4 and free T4 (FT4) levels in premature newborns vary according to gestational age in the first days of life. T4 and FT4 concentrations decline to the lowest level between ten and fourteenth days after birth. This situation is more severe with low gestational week and birth weight (BW) [4]. In term infants (37–42 weeks’ gestation) serum T4 levels characteristically increase in the first week of life whereas in infants born prematurely, and especially those below 30 weeks’ gestation, may decrease transiently resulting in a period of hypothyroxinaemia [5, 6].

TSH screening is the most sensitive test for primary CH detection. However, a unique form of CH characterized by a delayed TSH elevation has been described in preterm infants [2]. The screening programme may not identify CH in premature infants due to delayed TSH elavation, therefore, rescreening is recommended for all preterm infants by Europen Society for Pediatric Endocrinology, on behalf of all pediatric endocrinologist societies worldwide [7]. Continuing debate exists regarding whether THOP is harmful to the developing brain. While there are studies showing the benefit of levothyroxine (L-T4) treatment, there are also studies reporting that the treatment does not make differences or even causes worse neurodevelopmental outcomes [8]. Although treatment of hypothyroxinemia is recommended in newborns below 28 week of gestational age (GA), there is currently no concencus on this issue [9]. In a recent randomized controlled study conducted by May et al., premature babies born earlier than 28 wk. were divided into two groups and one group was given thyroxine from the first days, and the control group was not given any treatment. Motor, language, and cognitive functions were found to be significantly higher in Bayley III development tests performed at 42 months in the group that received the thyroxine treatment [10].

Postpartum TSH and thyroid hormone peaks observed in term newborns are not so evident in preterms. Especially below 30 weeks’ gestation, TSH increase is late and weak, while T4 and FT4 levels remain low [11]. In premature newborns, physiologicaly low thyroid hormones can be explained by: blunted phsyiolgic hyperthyroidim (correlated with gestational age), increase of thyroid hormone demand (thermogenesis, cardiac and skeletal muscle functions), deficiencies in iodine metabolism, immaturity of hypothalamo-pituitary-thyroid axis, insufficient response of thyroid gland to TRH, low T4 conversion from T3 and early interruption of maternal T4 transport [12, 13]. In addition to these factors, thyroid functions may also be suppressed due to medications that are commonly used in premature infants (dopamine, dexamethasone), disorders, such as respiratory distress syndrome, infections, necrotizing enterocolitis, patent ductus arteriosus, malnutrition, chorioamnionitis, as well as iodine deficiency or excess [12, 14–16]. Thus THOP can be observed due to multiple factors in premature newborns. This study aimed to evaluate the factors associated with THOP.

## Method

### Participants and Datas

This is a single center, retrospective, case-control study. The THOP and control groups (euthyroid) of premature newborns with similar gestational age and closest date of birth time were compared. File records of 538 newborns who had been admitted to Istanbul University-Cerrahpasa Faculty of Medicine Neonatal Intensive Care Unit (NICU) between January 2014–December 2019 were retrospectively analyzed. Recruitment criteria: premature newborns between 24 and 34 GA, without multiple major congenital anomaly and whose serum thyroid function test were done between the 10th and 20th days of postnatal life. Cases with major multiple organ anomalies excluded from the study (two cases had hydrops fetalis, one case had trisomy 18, two cases had multiple extremity anomalies and ventriculomegaly, two cases had kidney and cardiac anomalies).

**Fig. 1:**
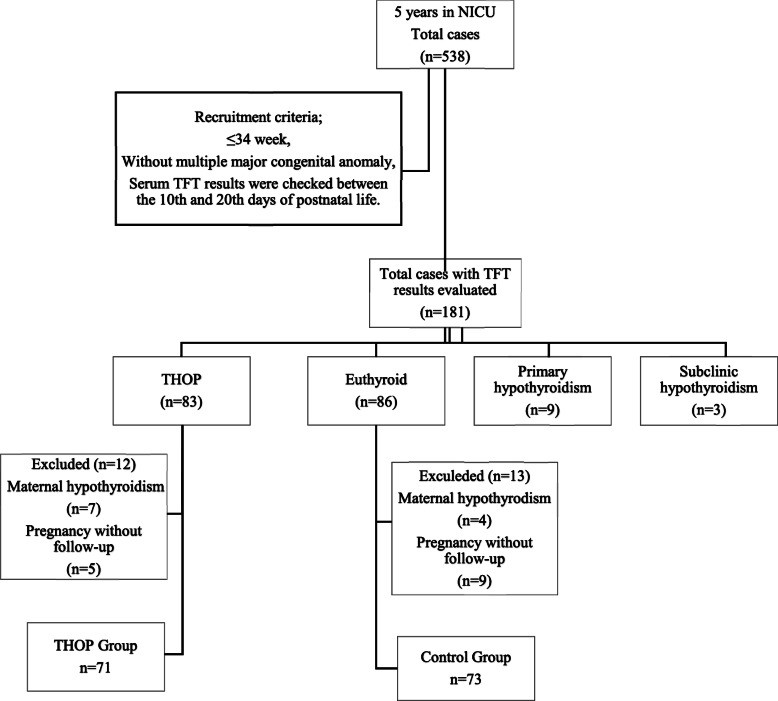
Study design. Abbreviations: TFT, thyroid function test; THOP, transient hypothyroxinemia of prematüre

During this 5-year retrospective study: THOP, euthyroid, primary hypothyroidism and subclinical hypothyroidism were diagnosed in 45.8% (*n* = 83), 47.5% (*n* = 86), 4.9% (*n* = 9) and 1.6% (*n* = 3), respectively. Infants of mothers with maternal hypothyrodism, pregnancy without follow-up was excluded from both THOP and euthyroid groups. Two groups were formed as the patient group (*n* = 71, THOP) and the control group (*n* = 73, euthyroid). The study design was planned as shown in Fig. 1.

Thirty six possible associated factors detected between birth to the time of blood samples collection (prenatal and postnatal parameters, medical treatments, clinical diagnoses and applications in NICU) were compared between the patient group with THOP (*n* = 71) and the control group with euthyroid infants (*n* = 73). Data including applied medical treatments and clinical applications were collected from the date of birth to the measurement of TSH and FT4.

Ethics approval was obtained from Istanbul University-Cerrahpaşa Faculty of Medicine Ethics Committee (reference no: 36423).

### Definitions

Serum free thyroxine (FT4) and TSH levels were measured using commercially available kits (Roche Elecys 2010, USA) by electrochemiluminescence (ECLIA) method. The thyroid hormone levels were assessed based on the weeks’ gestational and postnatal age references. In the second postnatal week following intervals were accepted to be the normal range for FT4: 1.45 ± 0.5 ng/dl for 23–27 week, 1.65 ± 0.4 ng/dl for 28–30 week, 1.98 ± 0.4 ng/dl for 31–34 week and the normal range for serum TSH: 3.9 ± 2.7 mU/L for 23–27 week, 4.9 ± 11.2 mU/L for 28–30 week, 3.8 ± 9.3 mU/L for 31–34 week were taken [1]. THOP was defined as a low FT4 (as per the age reference interval) and low or normal TSH (as per the age reference interval) levels [8]. Serum TSH and FT4 measurements were performed between the 10th and 20th days of postnatal life.

Bronchopulmonary dysplasia (BPD) was defined as the development of concomitant parenchymal lung injury requiring treatment with oxygen > 21% and/or positive pressure at postmenstrual gestational age at 36 weeks or at postnatal 56 days or at discharge (which one is earlier) [17]. Echocardiography evaluation was performed by pediatric cardiologist, patent ductus arteriosus (PDA), and other congenital heart diseases (CHD) were recorded. In our practice, both clinical findings and echocardiographic measurements were used to diagnose a hemodynamically significant PDA [18]. Respiratory Distress Syndrome (RDS) was diagnosed in infants who with signs in the chest radiograph (low lung volumes and reticulogranular appearance in the lung fields, with or without air bronchograms) and in those requiring oxygen to maintain target saturation, requiring surfactant treatment. Premature newborns who received mechanical ventilation or non-invasive ventilation support for more than 24 h were considered to receive ventilation support. Sepsis was diagnosed with positive blood cultures. Severe intraventricular hemorrhage (IVH) was defined as grade 3 or 4, while the presence of severe retinopathy of prematurity (ROP) was defined as grade 3 and beyond based on the International ROP classification [19, 20]. Fetal growth restriction (FGR) is defined as the failure of the fetus to achieve its genetically determined growth potential [21]. The cases whose weight was below the 10th percentile in antenatal percentile follow-ups were defined as FGR by a specialist in perinatology. Small for gestational age (SGA) is defined by birth weight below the 10th percentile for gestational age [22]. Central catheterisation was performed in two ways: umbilical arterial/venous cathether and Peripherally Inserted Central Catheter (PICC).

### Statistical methods

The SPSS v.21 (SPSS Inc., Chicago, IL, USA) software was used for the statistical analyzes. The compatibility of the data with normal distribution was evaluated by the descriptive statistics (mean, standard deviation) and Kolmogorov Smirnov Test. Continuous variables were shown with mean ± standart deviation and median (25th - 75th quarters), and categorical variables with frequency and percentage (%). The Mann-Whitney U test was used to compare the groups in terms of continuous variables. The Chi-squared test and Fisher’s exact test were used, where appropriate, in the analysis of the categorical variables. Possible associated factors with THOP which have *p* < 0.2 were included in univariate logistic regression analysis. The results were given as the Odds Ratio (OR) and 95% confidence intervals (95% CI). The level of significance was taken as *p* < 0.05.

## Results

Characteristics of the THOP and control groups are presented in Table 1. As expected, the FT4 levels were lower in the THOP than the control group (1.00 ± 0.24 and 1.55 ± 0.28 ng/dl, respectively) (*p* < 0.001), while there was no significant difference between the TSH levels (4.76 ± 3.36 and 4.32 ± 2.60 mU/L, respectively) (*p* = 0.556). Mean birth weight was lower in the THOP group (1306,52 ± 446,44 g) compared to the control group (1544,93 ± 498,64 g) (*p* < 0.001) (Table 2).

**Table 1:** Demographic Features and Laboratory Findings of THOP Patients and Controls

	THOP group	Control group	*P* value
Participants, n	71	73	
Male gender	41 (58)	34 (47)	0.180
GA (wk)	29.7 ± 2.48 (24–34)	30.5 ± 2.30 (26–34)	0.606
Measurement day	11.9 ± 2.22 (10–20)	12.3 ± 2.92 (10–20)	0.948
Measurement GAA (wk)	31.4 ± 2.42 (26–36)	32.2 ± 2.18 (28–36)	0.064
FT4 (ng/dl)	1.00 ± 0.24 (0.31–1.58)	1.55 ± 0.28 (0.97–2.54)	**< 0.001**
TSH (mU/L)	4.76 ± 3.36 (0.25–10.64)	4.32 ± 2.60 (0.73–13.3)	0.556

Data are given as numbers and (%), after decimal rounded to the greater side. Other data are given as mean ± standard deviation (min-max). When the THOP group and control group cases were compared, FT4 was lower as expected (*p* < 0.05, dark stained)

Abbreviations: *THOP* transient hypothyroxinemia of prematüre; *GA* gestational age; *GAA* gestation-adjusted age

**Table 2:** Comparison of possible associated factors with THOP between patients and controls

Risk factors	THOP group	Control group	*P* value
Participants, (n)	71	73	
BW (g)	1306,52 ± 446	1544,93 ± 498	**< 0.001**
Apgar score < 7 (5th minute)	34 (48)	24 (33)	**0.066**
Apgar score ≥ 7 (5th minute)	37 (52)	49 (67)	
Delivery type (C/S)	63 (89)	67 (92)	0.537
FGR	19 (27)	12 (16)	**0.132**
SGA	15 (21)	4 (5)	**0.006**
*PRENATAL*			
Maternal smoking	5 (7)	5 (7)	1.000
IVF	8 (11)	7 (10)	0.955
GDM	6 (9)	13 (18)	**0.097**
Gestational hypertension	20 (28)	21 (29)	1.000
Prenatal betamethasone treatment	47 (66)	48 (66)	1.000
*MEDICINE*			
Vancomycin+amikacin	9 (13)	5 (7)	0.238
Ampicillin+gentamicin	36 (51)	41 (56)	0.511
Surfactant	39 (55)	34 (46)	0.403
Caffeine	59 (83)	57 (78)	0.582
Dobutamine	8 (11)	3 (4)	**0.106**
Dopamine	17 (24)	13 (18)	0.365
Midazolam	23 (32)	21 (29)	0.637
Fentanyl	4 (6)	2 (3)	0.385
Paracetamol	3 (4)	2 (3)	0.679
Ibuprofen	9 (13)	4 (6)	**0,132**
Fluconazole prophylaxis	34 (48)	32 (44)	0.626
*CLINICAL-DIAGNOSIS*			
RDS	39 (55)	34 (56)	0.403
IVH	24 (34)	12 (16)	**0.022**
Pneumothorax	3 (4)	4 (5)	0.726
PDA	12/59 (20)*	6/57 (11)*	**0.144**
CHD	15/59 (25)*	5/57 (9)*	**0.018**
ROP	3 (4)	1 (1)	0.448
BPD	26 (37)	16 (22)	**0.052**
Hypoglycemia	21 (30)	23 (31)	0.802
Sepsis	7 (10)	9 (12)	0.837
*TRANSFUSIONS*			
ES transfusion	18 (25)	13 (18)	0.271
TS transfusion	5 (7)	5 (7)	0.964
FFP transfusion	23 (32)	19 (26)	0.401
*CLINICAL APPLICATIONS*			
Phototherapy	66 (93)	68 (93)	0.964
Central catheter	57 (80)	57 (78)	0.905
Ventilator support	31 (43)	28 (38)	0.517
NIV support	48 (68)	54 (74)	0.511

Data are given as numbers and (%), after decimal rounded to the greater side. Parameters ​​with p < 0.2 were included in univariate logistic regression analysis (dark stained ones)

*Data are given as cases / total cases with echocardiographic examination

Abbreviations: *THOP* transient hypothyroxinemia of prematüre; *BW* birth weight; *C/S* caesarean; *FGR* fetal growth retardation; *SGA* small for gestational age; *IVF* in vitro fertilization; *GDM* gestational diabetes; *IVH* intraventricular hemorrhage; *PDA* patent ductus arteriosus; *CHD* congenital heart disease; *ROP* premature retinopathy; *BPD* bronkopulmonary dysplasia; *ES* erirtosit suspension; *TS* thrombcyte suspension; *FFP* fresh frozen plasma; *NIV* non invasive ventilation

In the THOP group, a total of 15 cases of CHD; 8 cases of atrial septal defect (ASD), 3 cases of ventricular septal defect (VSD), 1 case of transposition of the great arteries (TGA), 1 case of coarctation of the aorta (CoA), 1 case of atrial septal aneurysm, 1 case of mitral valve prolapse were detected. In the control group, 4 patients had ASD and 1 VSD. In THOP group, CHD was statistically significantly higher than control group (*p* = 0.018, Table 2).

When 36 associated factors were compared between the THOP patients and the control group in univariate analysis: 5th minute Apgar scores, delivery type, FGR, in vitro fertilization (IVF), maternal smoking, gestational hypertension and diabetes, prenatal betamethasone treatments, central catheterisations, sepsis, pneumothorax, PDA, drug history (vancomycin+amikacin, ampicillin+gentamisin, caffeine, dopamine, dobutamine, fentanyl, midazolam, paracetamol, ibuprofen, fluconazole), phototherapy, development of hypoglycemia, RDS, BPD and ROP, erythrocyte suspension (ES), thrombocyte suspension (TS) and fresh frozen plasma transfusions, mechanical ventilator and noninvasive ventilation support were not statistically significant (Table 2). BW was lower and incidences of IVH, SGA and CHD were higher in the THOP group (*p* < 0.05, Table 2). Possible associated factors with THOP which have *p* < 0.2 were included in univariate logistic regression analysis (fetal growth retardation, gestational diabetes, 5th minute Apgar score, BW, SGA, dobutamine, ibuprofen, IVH, bronchopulmonary dysplasia, CHD) (*p* < 0.200) were analyzed. The CHD (*p* = 0.007, odds ratio [OR]:4.9, 95% confidence interval [CI]: 1.5–15.8), BW (*p* = 0.004, OR:0.999, 95% CI: 0.9–1.0) and SGA (*p* = 0.010, OR:4.6, 95% CI: 1.4–14.7) were found to be factors associated with THOP (*p* < 0.05, Table 3). As seen in the Table 3, the risk of THOP was found to be increased 4.9 and 4.6 times more in premature infants diagnosed with CHD and SGA, respectively. It was also determined that each one houndred gram increase in BW reduced the THOP risk by 10% (Table 3).

**Table 3:** Factors associated with THOP determined by univariate logistic regression analysis

Factors	*P* Value	OR	95% CI
FGR (Ref. Absent)	0.135	1.857	0.825–4.183
GDM (Ref. Absent)	0.104	0.426	0.152–1.192
Apgar score, 5th min. (Ref. ≥ 7)	0.068	1.876	0.955–3.684
Birth weight (gram)	**0.004**	0.999	0.998–1.000
SGA (Ref. Absent)	**0.010**	4.621	1.452–14.708
Dobutamine (Ref. Absent)	0.120	2.963	0.753–11.659
Ibuprofen (Ref. Absent)	0.142	2.504	0.734–8.539
IVH (Ref. Absent)	0.178	1.829	0. 776–4.356
BPD (Ref. Absent)	0.054	2.058	0.987–4.294
CHD (Ref. Absent)	**0.007**	4.930	1.535–15.840
PDA (Ref. Absent)	0.151	2.170	0.754–6.246

Birth weight, SGA and detection of CHD were found to be statistically significant (*p* < 0.05, dark stained ones)

Abbreviations: *FGR* fetal growth retardation; *GDM* gestational diabetes; *BW* birth weight; *SGA* small for gestational age; *IVH* intraventricular hemorrhage; *BPD* bronkopulmonary dysplasia; *CHD* congenital heart disease; *Ref* reference category

## Discussion

In this study, The THOP and control groups (euthyroid) of premature newborns born at the same time and with the most similar gestational age were compared. Low birth weight, SGA and CHD were found to be as associated factors with THOP. These three associated factors are not affected by the gestational age, the severity of the illness and the medical and clinical treatment practices applied. It is already shown in the literature that the frequency of hypothyroxinemia changes in proportion to gestational age [23]. Especially in sick infants, if hypothyroxinemia is not noticed and followed up, it may be missed in TSH screening for congenital hypothyroidism [16]. In addition, in a recent randomized and controlled study by May et al., showed a better neurological development in preterm infants under 28 weeks of gestation who had a hypothyroxinemia and who received a treatment [10]. Therefore, timely recognition and follow-up of transient hypothyroxinemia in preterm infants is important.

### THOP and SGA

In our study, interestingly although THOP group had similiar gestational age with the control group, had a low birth weight in THOP group. Consequently in this study, incidence of SGA was higher in the THOP group and increased the risk of the THOP 4.6 times. Each one hundred gram increase in BW reduced the THOP risk by 10%. We speculate that being SGA may result in further deficit in storage and adaptive mechanisms. A recent study by Chunhua et al., supported our results that SGA in preterms may be associated with thyroid dysfunction [24]. A comprehensive study by Bagnoli et al. showed that similar to our results, preterm SGA neonates had lower FT4 compared to preterm AGAs and TSH levels were similar [25]. It is generaly accepted that hypothyroxinemia is usually transient in preterm SGAs and is caused by placental hypoxia and delayed maturation of the thyroid gland [26]. In addition, some studies underlined that hypothyroxinemia in preterm SGAs might be attributed to nutritional deficiency and might be reversible with the regulation of nutrition [27, 28]. Similar to the literature, the results of our study suggested that thyroid function tests should be followed more closely in both preterm and SGA newborns.

### THOP and CHD

It is already shown that thyroid and cardiac disorders can be associated [29]. Congenital heart diseases (5.5%) are frequently associated with congenital hypothyroidism sugggesting common genetic mechanisms involved in thyroid and heart development [30]. The association of CHD and thyroid disorder in Down syndrome, which is a genetic disorder, has been well defined [31]. In the recent study of HJ Lee et al., it was found that the coexistence of CHD and transient thyroid disorders is approximately 50% [32]. In a study with 76 preterm infants hospitalized in neonatal units, cardiovascular disease was significantly higher in THOP [33]. In a comprehensive study by Sadia Malik et al., evaluating the relationship between SGA and CHD, CHD was found to be twice as high in infants with SGA compared to the control group [34]. Decreased birth weight, SGA and CHD may have common pathogenetic mechansims that are associated with THOP. Similarly, in this study, hypothyroxinemia was found with a higher rate in preterms with SGA and CHD. It is well known in the literature that the combination of CHD and SGA increases mortality and morbidity [35]. Therefore, close follow-up of thyroid function tests is more important in preterms with CHD and SGA.

### THOP and prenatal-postnal conditions

Some studies have shown that in preterms thyroid functions are affected by postpartum drugs and some perinatal conditions. Drugs frequently used in premature infants (dopamine, dexamethasone), respiratory distress syndrome, infections, disorders such as necrotizing enterocolitis, patent ductus arteriosus, malnutrition, chorioamnionitis, iodine deficiency or overload may suppress thyroid functions [23, 36–39]. It should be noted that gestational age is inversely correlated with the severity of problems in the premature newborns which may be associated with more intense medical and invasive treatments. Thus, it is difficult to evaluate whether an associated factor is the consequence of immaturity or the medical treament. Previous studies reported that surfactant, dopamine, glucocorticoids and erythrocyte transfusion increase the risk of THOP [40–43]. Most of these studies evaluating the associated factors for hypothyroxinemia belong to the past years and are studies with a smaller number of cases compared to our study.

The medications are used widely as gestational age decrease and severity of the disorder increases. In a study with very low birth weight newborns, it was shown that the negative effect of dopamine and dobutamine use on thyroid function rapidly resolved after treatment was discontinued [44]. There are some studies showing that the suppression of thyroid functions has decreased in parallel with the decrease in RDS severity due to the development of prenatal care, especially the widespread use of antenatal steroids and early surfactant treatment in recent years [23]. In a study by Lay et al., no relationship was shown between THOP and PDA, IVH, antenatal steroid use, ROP, Apgar scores, and sepsis [4]. In different pathologies like respiratory distress syndrome, PDA, sepsis, intracranial hemorrhage and necrotizing enterocolitis, it is claimed that their effects on serum thyroid hormone levels are mediated in part by acute inflammatory cytokines [23]. In our study, differently from previous studies RDS, PDA, sepsis, IVH, dopamine, dobutamine and erythrocyte suspension transfusion were not found to be the associated factors for THOP. The reason for these different results in our study, may be due to less needed treatments and their temporary effects with the widespread use of surfactants and antenatal steroids in recent years and the decrease in the severity of diseases in neonatal units.

In this study, serum TFT results between ten and twenty days of life were evaluated according to the postnatal and gestational age reference values (1). However, there is no TFT level defined as “low or normal” and there is no consensus on the timing of measurements. The strength of this study is that it is one of the few studies [11, 14, 40–44] evaluating so many parameters associated with THOP in premature newborns. On the other hand our study have some limitations as being a single center and retrospective study. A multi center and prospective study could create more meaningful results.

In conclusion, although some treatment practices could have direct effects on the pituitary-thyroid axis in relation to the severity of neonatal clinical conditions, none of these were found to be an associated factor with THOP in our study. CHD and SGA were found to be as associated factors with THOP.

## Data Availability

The datasets generated and/or analysed during the current study are not publicly available due to our hospital policy but are available from the corresponding author on reasonable request.

## Abbreviations

THOP: transient hypothyroxinemia of prematüre
BW: birth weight
C/S: caesarean
FGR: fetal growth retardation
SGA: small for gestational age
IVF: in vitro fertilization
GDM: gestational diabetes
IVH: intraventricular hemorrhage
PDA: patent ductus arteriosus
CHD: congenital heart disease
ROP: premature retinopathy
BPD: bronkopulmonary dysplasia
ES: erirtosit suspension
TS: thrombcyte suspension
FFP: fresh frozen plasma
NIV: non invasive ventilation
